# Safety, pharmacokinetics, and preliminary efficacy of E6201 in patients with advanced solid tumours, including melanoma: results of a phase 1 study

**DOI:** 10.1038/s41416-018-0099-5

**Published:** 2018-06-05

**Authors:** Raoul Tibes, Mitesh J. Borad, Corina E. Dutcus, Larisa Reyderman, Kevie Feit, Andrew Eisen, David A. Verbel, Daniel D. Von Hoff

**Affiliations:** 1 Laura & Isaac Perlmutter Cancer Center, New York University School of Medicine, New York, NY USA; 2 Mayo Clinic, Scottsdale Campus, Scottsdale, AZ USA; 3 Eisai Inc., Woodcliff Lake, NJ USA; 4 Translational Medicine Consulting, Rockville, MD USA; 5 Translational Genomics Research Institute, Phoenix, AZ USA; 6 HonorHealth, Scottsdale, AZ USA

**Keywords:** Cancer, Metastasis

## Abstract

**Background:**

This phase 1 first-in-human study aimed to determine the maximum-tolerated dose (MTD), dose-limiting toxicities, and safety of E6201, and to establish recommended dosing in patients with advanced solid tumours, expanded to advanced melanoma.

**Methods:**

Part A (dose escalation): sequential cohorts received E6201 intravenously (IV) over 30 min (once-weekly [qw; days (D)1 + 8 + 15 of a 28-day cycle]), starting at 20 mg/m^2^, increasing to 720 mg/m^2^ or the MTD. Part B (expansion): patients with *BRAF*-mutated or wild-type (WT) melanoma received E6201 320 mg/m^2^ IV over 60 minutes qw (D1 + 8 + 15 of a 28-day cycle) or 160 mg/m^2^ IV twice-weekly (D1 + 4 + 8 + 11 + 15 + 18 of a 28-day cycle; *BRAF*-mutated only).

**Results:**

MTD in Part A (*n* = 25) was 320 mg/m^2^ qw, confirmed in Part B (*n* = 30). Adverse events included QT prolongation (*n* = 4) and eye disorders (*n* = 3). E6201 exposure was dose-related, with PK characterised by extensive distribution and fast elimination. One patient achieved PR during Part A (*BRAF*-mutated papillary thyroid cancer; 480 mg/m^2^ qw) and three during Part B (2 *BRAF*-mutated melanoma; 1 *BRAF*-WT melanoma; all receiving 320 mg/m^2^ qw).

**Conclusions:**

An intermittent regimen of E6201 320 mg/m^2^ IV qw for the first 3 weeks of a 28-day cycle was feasible and reasonably well-tolerated in patients with advanced solid tumours, including melanoma with brain metastases, with evidence of clinical efficacy.

## Introduction

The Raf/Ras/MEK/extracellular signal-related kinase (ERK) signalling pathway regulates multiple cellular functions, and activating mutations commonly affect this pathway in human cancers.^1,2^ *Ras* mutations occur in up to 30% of cancers, and mutations in B-type Raf (*BRAF*) kinase (an upstream kinase of mitogen-activated protein kinase/extracellular signal-regulated kinase kinase-1 [MEK1]), although less common, are prevalent in specific malignancies.^1,3^ For example, somatic missense mutations in *BRAF* occur in 67% of malignant melanomas, 12% of colorectal cancers, and 100% of hairy cell leukaemias.^3,4^ Most mutated BRAF proteins have elevated kinase activity, leading to activation of MEK1 and 2, which trigger ERK phosphorylation and activate downstream pathways.^3,5,6^ MEK has been explored as a therapeutic target and, in 2013, trametinib (Mekinist^®^, Novartis), a MEK1/MEK2 inhibitor, was the first MEK inhibitor approved to treat patients with unresectable/metastatic malignant melanoma with *BRAF* mutations (V600E or V600K).

E6201 (Eisai Inc., Woodcliff Lake, NJ) is a synthetic analog of a naturally occurring product of the fungus *Curvularia verruculosa*.^7^ It is a potent inhibitor of MEK1 and mitogen-activated protein kinase/extracellular signal-regulated kinase kinase kinase-1 (MEKK1), and other kinases with a role in cancer development, including Src tyrosine kinases.^7–11^ Cancers with elevated MEK1 activity, including those with an upstream activating *BRAF* mutation, may be therapeutic targets for E6201.^12,13^ Preclinical studies have demonstrated the ability of E6201 to inhibit growth and induce cell death in *BRAF*-mutated human cancer cell lines and xenografts, including melanoma.^13–15^

This phase 1, multicentre, open-label, first-in-human study of E6201 (Eisai Inc.; www.clinicaltrials.gov, NCT00794781) was conducted in 2 parts. Part A, a dose-escalation study in patients with advanced solid tumours, determined the maximum-tolerated dose (MTD) and dose-limiting toxicities (DLTs) of once-weekly E6201. Part B, an expansion in patients with advanced *BRAF*-mutated or wild-type (WT) melanoma, evaluated the safety and tolerability of both once-weekly and twice-weekly E6201, establishing optimal dosing in this population. Additionally, the study evaluated pharmacokinetics (PK) and preliminary clinical efficacy of E6201, explored pharmacodynamics (PD) and correlation with tumour mutational status, and explored potential surrogate biomarkers (to be published separately).

## Materials and methods

### Patient population

Eligible patients included adults with histologically and/or cytologically confirmed metastatic solid tumours (Part A) or metastatic *BRAF*-mutated or WT melanoma (Part B) that had progressed after treatment or for which no standard effective therapies were available. Those who had undergone prior surgery or recent treatment for their disease, or had a history of cardiac problems, were excluded (see Supplementary Material).

### Study design and treatment

The primary objectives of this phase 1 study were to evaluate the safety, tolerability, and MTD of once-weekly (Schedule I) and twice-weekly (Schedule II) dosing. Secondary objectives included evaluation of the PK profile of E6201. During Part A, sequential cohorts of 3–6 patients received E6201 as a 30-min intravenous (IV) infusion once-weekly for the first 3 weeks of a 28-day cycle (Days 1, 8, and 15), starting at 20 mg/m^2^ and increasing in 100% increments to 320 mg/m^2^. Dosing then escalated in 50% increments or less, up to 720 mg/m^2^ (to limit exposure to Captisol^®^ excipient in the E6201 formulation), or until MTD was determined. Toxicity was managed by dose interruption, reduction, and/or discontinuation. Only Cycle 1 adverse events (AEs) were evaluable as DLTs. Based on AEs reported at 480 mg/m^2^ and 400 mg/m^2^, the protocol was amended to include up to 6 additional toxicity-evaluable patients at 320 mg/m^2^, to confirm this as the MTD. All ongoing patients could continue at 320 mg/m^2^, regardless of initial starting dose.

In Part B, an adaptive study design was planned to define an optimal dosing schedule. Two schedules were evaluated: Schedule I (once-weekly dosing at the MTD, 320 mg/m^2^, on Days 1, 8, and 15 of a 28-day cycle) and Schedule II (twice-weekly dosing, on Days 1, 4, 8, 11, 15, and 18 of a 28-day cycle, starting at 160 mg/m^2^, based on the Schedule I MTD). One dose escalation (from 160 mg/m^2^ to 320 mg/m^2^) was allowed in Schedule II for MTD determination. E6201 infusion time was increased from 30 to 60 min in Part B, to decrease maximum observed plasma concentration (*C*_max_) and reduce the potential for QTc prolongation. Toxicity was managed as before. Up to 30 patients with *BRAF*-mutated melanoma and up to 29 patients with *BRAF*-WT melanoma were to be enrolled in Schedule I. Up to 30 patients were to be enrolled into Schedule II once the MTD was confirmed. Based on the efficacy observed in Part A, Schedule II was initiated only in patients with *BRAF*-mutated melanoma. Patients could continue to receive E6201 as long as there was evidence of clinical benefit (extension phase).

The study was conducted from July 2008 to August 2011 (database cut-off) in the United States, in accordance with the Declaration of Helsinki. It was approved by an Institutional Review Board/Independent Ethics Committee at each study centre and in accordance with an assurance filed with and approved by the U.S. Department of Health and Human Services. All patients provided signed informed consent prior to trial entry.

### Safety assessments

Safety was assessed by monitoring AEs, serious AEs (SAEs), laboratory parameters, vital signs, 12-lead electrocardiograms (ECGs), Eastern Cooperative Oncology Group performance status, and symptom-directed physical and neurologic examination findings. AEs were graded by investigators based on the National Cancer Institute Common Terminology Criteria for Adverse Events, version 3.^16^

### Pharmacokinetic analysis

Samples were collected for PK analyses of E6201 and its active metabolite ER-813010 using a validated assay. In Part A, blood was collected on Cycle 1, Days 1 and 15 at specified intervals prior to, during, and postinfusion (5 min–8 h), and on Days 2, 3, 16, and 17 (24 and 48 h postinfusion). Urine was collected on Cycle 1, Day 1 preinfusion, at 0–8 h, and 8–24 h. In Part B, blood was collected on Cycle 1, Day 1, prior to, during, and at 1–24 h postinfusion. Pharmacokinetic parameters were calculated using noncompartmental analysis.

### Efficacy assessments

Efficacy was assessed by tumour response determined by investigator review of computed tomography/magnetic resonance imaging scans using Response Evaluation Criteria in Solid tumours version  1.0 (RECIST).^17^

### Statistical analyses

Efficacy and safety were evaluated using the safety analysis population (all patients who received ≥1 E6201 infusion with ≥1 subsequent safety assessment).

## Results

### Patient characteristics and disposition

Demographic and baseline characteristics are summarised in Table 1.

**Table 1:** Demographics and baseline characteristics (safety population)

	Part A (*n* = 25)	Part B (*n* = 30)
Median age (range), years	66 (44–87)	56 (28–79)
Gender, *n* (%)		
Male	10 (40.0)	15 (50.0)
Female	15 (60.0)	15 (50.0)
ECOG performance status, *n* (%)		
0	14 (56.0)	9 (30.0)
1	11 (44.0)	21 (70.0)
Tumour type, *n* (%)		
Melanoma	3 (12.0)	30 (100)
Colon	5 (20.0)	0
Breast	2 (8.0)	0
Prostate	2 (8.0)	0
Gall bladder	2 (8.0)	0
Pancreas	2 (8.0)	0
Papillary thyroid	2 (8.0)	0
Other^a^	7 (28.0)	0
Tumour mutational status, *n* (%)		
*BRAF*-mutated/*BRAF*-WT	3 (12.0)/15 (60.0)^b^	23 (76.7)/7 (23.3)
*KRAS*-mutated/*KRAS*-WT	3 (12.0)/15 (60.0)^b^	Not collected in Part B
Number of previous therapies for locally advanced or metastatic disease, *n* (%)		
0	3 (12.0)	7 (23.3)
1	3 (12.0)	12 (40.0)
2	7 (28.0)	7 (23.3)
3	6 (24.0)	2 (6.7)
4	3 (12.0)	2 (6.7)
≥5	3 (12.0)	0
Type of prior therapy, *n* (%)		
Radiotherapy	10 (40.0)	15 (50.0)
Neoadjuvant	2 (8.0)	0
Adjuvant	8 (32.0)	8 (26.7)

^a^Duodenal, GIST, rectal, GE junction, hepatobiliary, thyroid, gastric carcinoid (*n* = 1 each).

^b^Mutation status based on archival tumour tissue, no sample/status unknown for 7 patients.

*GE* gastroesophageal, *GIST* gastrointestinal stromal tumour, *WT* wildtype

Twenty-five patients were enrolled in Part A; 19 (76%) patients completed Part A (continued E6201 treatment until clinical or radiologic progression); and 6 (24%) discontinued study treatment prematurely. Part B enrolled 30 patients; at data cut-off (01 Aug 2011), 19 (63.3%) had completed study treatment, 6 (20%) had discontinued prematurely, and 5 (16.7%) continued study treatment/follow-up. Part B was terminated early due to futility based on response data. However, one patient with *BRAF*-mutated melanoma (a patient with multiple metastases, including brain metastases) was still receiving E6201 in April 2017 because of an outstanding response to therapy.

### Maximum-tolerated dose

In Part A, the MTD of E6201 as a 30-min IV infusion once-weekly for 3 weeks of a 28-day cycle was determined to be 320 mg/m^2^. At 480 mg/m^2^, 3 patients had DLTs. These DLTs comprised QTc prolongation (QTcF > 500 ms), grade 4 confusion, and QTcF increase from baseline > 60 ms. Dosing of E6201 was reduced to 400 mg/m^2^, but 1 patient experienced central nervous system (CNS) toxicity (grade 1 dizziness). This AE did not meet DLT criteria, but was treated conservatively as a DLT because this was the second patient with CNS toxicity. The events in the 400 mg/m^2^  and 480 mg/m^2^ cohorts suggested a possible effect of E6201 on the membrane repolarisation process manifested in either central neurotoxicity or delayed cardiac repolarisation. Additional patients were enrolled at a dose of 320 mg/m^2^. One patient (of *n* = 7 at this dose) experienced a DLT (QTcF increase from baseline > 60 ms) and grade 2 CNS toxicities (slurred speech, blurred vision, dizziness).

At the time of data cut-off for Part B, no additional DLTs were observed, confirming the MTD as 320 mg/m^2^ once-weekly for 3 weeks of a 28-day cycle, administered as a 60-min infusion.

### Safety

All patients reported ≥1 treatment-emergent AE (TEAE); most commonly nausea in Part A (9 (36%) patients) and fatigue in Part B (11 (36.7%) patients). The incidence of the most common TEAEs during the study is summarised by dose in Supplementary Table S1. Eleven (44.0%) patients in Part A and 19 (63.3%) in Part B had ≥ 1TEAE assessed by the investigator as treatment-related. No dose-related or mutation status-related trends were observed.

Grade 3/4 TEAEs were reported in 11 (44.0%) patients in Part A and 15 (50.0%) in Part B. In Part A, the most common grade 3/4 AEs were abdominal pain, hyperbilirubinemia, ECG QT prolonged, and syncope (each reported by 2 (8.0%) patients). The majority were considered unrelated to study medication. Only 1 of these AEs was grade 4 (abdominal pain; considered possibly related to E6201 and unresolved despite treatment, so the patient discontinued the study). The most common grade 3/4 AEs in Part B were abdominal pain and dyspnea (each reported by 2 (6.7%) patients), none of which were grade 4 or considered related to E6201.

Four patients died during the study. In Part A, 1 patient in the 320 mg/m^2^ group died (post-Cycle 1) as a result of abnormal hepatic function considered possibly related to E6201. In Part B, 2 patients in the *BRAF*-mutated 320 mg/m^2^ once-weekly group died, 1 as a result of metastatic malignant melanoma and 1 due to an altered level of consciousness (accompanied by disease progression on tumour assessment). Both patients had CNS metastases treated with radiation prior to the study. One patient in the *BRAF*-mutated 160 mg/m^2^ once-weekly group died, as a result of multisystem organ failure. Additionally, one patient in Part B (*BRAF*-mutated 320 mg/m^2^ once-weekly group) had a SAE with a fatal outcome after study discontinuation (worsening dyspnea due to disease progression). No deaths in Part B were considered related to E6201. Treatment-emergent SAEs other than death were reported in 8 (32.0%) patients in Part A and 14 (46.7%) in Part B. Two patients in Part A and 4 in Part B discontinued treatment due to SAEs. The majority of SAEs were considered unrelated to E6201.

The incidence of ocular toxicity was low. In Part A, two patients reported grade 1/2 eye disorders. One patient in the 160 mg/m^2^ dose group had increased lacrimation (possibly related to E6201), while one patient in the 320 mg/m^2^ dose group reported blurred vision (possibly related) and visual impairment (unrelated; grade 1 photophobia lasting 2 days, recovered without treatment with no re-occurrence, not serious, no change to E6201 dosing). In Part B, one patient in the *BRAF*-mutated 320 mg/m^2^ once-weekly group reported grade 1 photophobia (possibly related to E6201).

During Part A, QTcF prolongation to 450–500 ms was seen in five patients (20.0%) across dose groups, and to >500 ms in one patient at the 480 mg/m^2^ dose in Cycle 2. QTcF increases of 30–60 ms and >60 ms from baseline were seen in five patients (20.0%) and two patients (8.0%), respectively. QT prolongation was reported as an AE by three patients (12.0%; Grade 2/3, all meeting DLT criteria and considered probably related to E6201).

QTcF increase from baseline was also reported during Part B. Prolongation to 450–500 ms was seen in four patients (13.3%) across dose groups. QTcF increases from baseline 30–60 ms were seen in seven patients (23.3%) and increases ≥60 ms were seen in two (6.7%) patients in the *BRAF*-mutated 320 mg/m^2^ once-weekly group during Cycle 2 and at two unscheduled visits. QT prolongation was reported as an AE by 1 (3.3%) patient in Part B (grade 3, possibly related to E6201, did not meet DLT criteria).

The incidence of other cardiac disorders was low. In Part A, one patient in the 320 mg/m^2^ group had grade 3 bradycardia (unrelated to E6201). In Part B, 1 patient in the *BRAF*-mutated 320 mg/m^2^ once-weekly group had grade 4 pericardial effusion (unrelated).

No new skin cancers were reported during the study. Additionally, there were no reported AEs of cardiomyopathy.

In this study, changes from baseline in laboratory parameters and vital signs were variable, generally small and clinically unimportant. There were no dose-related or mutation status-related trends.

### Pharmacokinetics

**Fig. 1:**
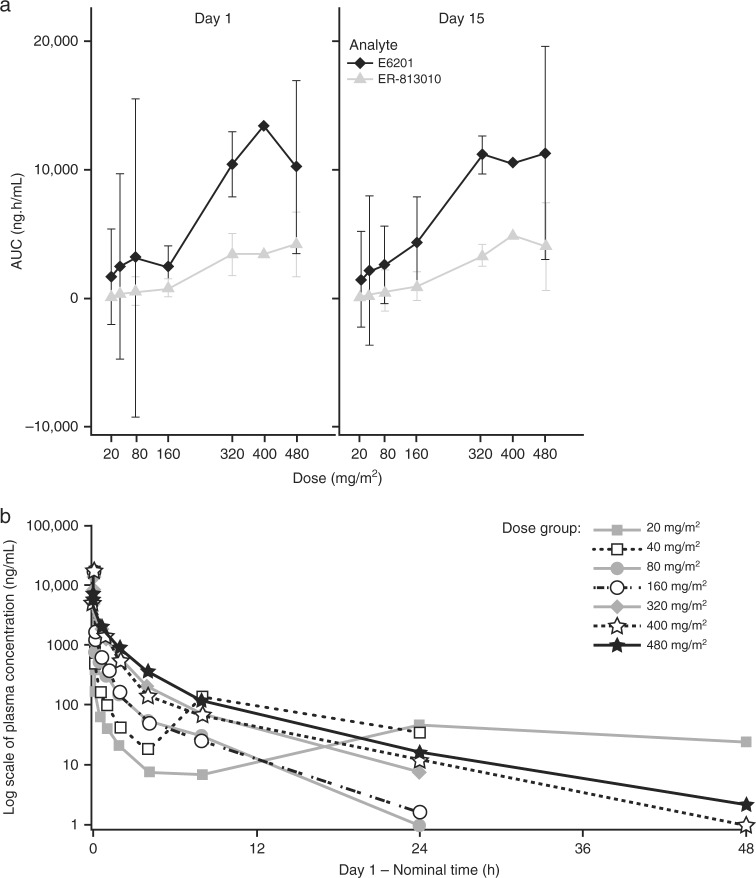
**a** Mean exposure versus E6201 dose for E6201 and its metabolite ER-813010 (Cycle 1). Points represent mean data, error bars represent standard deviation. *AUC* area under the curve. **b** Mean E6201 plasma concentration over time on Cycle 1 Day 1

E6201 concentration peaked by mid-to-end of infusion (0.25–0.5 h) and then declined in a multi-exponential manner (Supplementary Table S2). Mean elimination half-life (*t*_1/2_) ranged from 1.6 to 6.8 h. Most patients had concentrations below the limit of quantitation 48 h post-end of infusion. Mean exposure (area under the curve [AUC] and *C*_max_) generally increased in a dose-related manner up to the 320 mg/m^2^ dose, with only incremental change at 400 mg/m^2^ on day 1, which was not observed on day 15 (Fig. 1a). Overall, exposure was highly variable: % coefficient of variation (%CV) of AUC values ranged from 12% to 250%. E6201 PK results on multiple dosing (Day 15), including the dose–exposure relationship, were comparable to those following a single dose (Day 1) (Fig. 1a; Supplementary Table S2). Lack of E6201 accumulation on Day 15 is consistent with its relatively short half-life.

E6201 PK results were characterised by extensive distribution (Fig. 1b). Mean clearance (CL) ranged from 49.8–184 L/h, consistent with liver blood flow (87 L/h). Mean *V*_ss_ values (80.1–275 L) were markedly higher that total body water (42 L), suggesting extensive distribution of E6201 in peripheral tissues. The fraction of E6201 excreted in the urine over a 24-hour collection interval was ≤0.21% (Supplementary Table S2). Renal clearance (CL_R_) values (1.65–137 mL/h) were markedly lower than the glomerular filtration rate (~7500 mL/h).

E6201 was rapidly metabolised to its active metabolite ER-813010. Maximum plasma ER-813010 concentration was achieved 0.5 h postdose. ER-813010 elimination paralleled that of E6201, indicating formation-limited elimination. The half-life of ER-813010 was less variable than that of E6201 and ranged from 3.08–9.47 h. Similar to E6201, ER-813010 did not accumulate on multiple dosing.

In the dose-expansion phase, the effect of *BRAF* mutant genotype on the PK of E6201 and ER-813010 was assessed. Mean plasma concentrations of both analytes generally displayed similar profiles over time for each dose and genotype, indicating that *BRAF* mutation, as expected, had no effect on PK properties.

### Efficacy

In Part A, there was one partial response (PR), in a patient with V600E *BRAF*-mutated papillary thyroid cancer (*KRAS*-WT, *PTEN* weakly reactive) receiving E6201 480 mg/m^2^, who had a response for four cycles. In Part B, there were two PRs in patients with V600E *BRAF*-mutated melanoma (primary tumour on the right leg, *KRAS*-WT, *AKT*-positive, *PTEN*-positive; primary tumour on right inguinal node, other mutation status unknown due to lack of tissue for testing), who had responses for >40 cycles (36.8 months), and one PR in a patient with *BRAF*-WT, *KRAS*-WT melanoma (primary tumour on nose) who had a response for more than 2 cycles (3.0 months); all three patients were receiving E6201 320 mg/m^2^ once-weekly. Of note, one patient with *BRAF*-mutated melanoma who achieved a PR had brain metastases that responded to treatment with E6201. Best overall response is summarised in detail by dose group in Supplementary Table S3. Supplementary Figure S1 presents changes in tumour size during the study, including those for responding patients.

In Part A, eight patients (32.0%) achieved stable disease (SD). Of these, five (20.0%) had SD for ≥4 cycles (3.7 months), including one patient in the 80 mg/m^2^ dose group with *BRAF*-WT uveal melanoma who had SD for 15 cycles. In Part B, nine patients (30.0%) achieved SD: seven of these patients had SD for ≥4 cycles (3.7 months), of whom five were in the *BRAF*-mutated 320 mg/m^2^ once-weekly dose group.

In Part B, 2/23 patients with *BRAF*-mutated melanoma (receiving 320 mg/m^2^ once-weekly) had a PR compared with 1/7 *BRAF*-WT patients. Similarly, 8/23 patients with *BRAF*-mutated melanoma had SD (seven receiving E6201 320 mg/m^2^ once-weekly), compared with 1/7 *BRAF*-WT patients. The disease control rate (DCR;  complete response + PR + SD) was 43.5% (10/23) in patients with *BRAF*-mutated melanoma and 28.6% (2/7) in patients with *BRAF*-WT melanoma. Response duration in Part B could not be assessed because no responders had experienced disease progression at the time of data cut-off.

## Discussion

In preclinical models, E6201 administration (on a Q4D x3 schedule) in *BRAF*-mutated human cancer xenograft models resulted in significant and prolonged anti-tumour activity lasting >20 days from first dose.^13^ MEK1 inhibition occurred only 8 h after dosing, and was sustained for 72 h postdose despite the short *t*_1/2_ of E6201 (~3–6 h).^11,18^ Anticipating a similarly delayed effect in humans, a weekly dosing regimen was proposed. As E6201 also inhibits other cancer-relevant kinases, the dose-escalation phase enrolled patients without stratification for *BRAF* mutational status. Thus, we could not explore the relative importance of E6201 inhibition of mutant *BRAF* versus Src family and other tyrosine kinases.

After dose escalation, the MTD of E6201 was determined to be 320 mg/m^2^ when administered as a 30-min IV infusion once-weekly for the first 3 weeks of a 28-day cycle. The same schedule was also confirmed as the MTD in the expansion part of the study, enrolling patients with both *BRAF*-mutated and *BRAF*-WT metastatic melanoma, with the infusion time increased to 60 min to reduce the potential for QTc prolongation.

E6201 appeared to be well-tolerated during the study. However, it may have an effect on cardiac repolarisation manifested by prolongation of the QT/QTc interval; this effect may be reduced when infusion time is increased from 30 to 60 min. During dose-escalation (30-min infusion), ECG QT prolongation was reported as an AE (grade 2 or 3) in three patients (12.0%), all meeting DLT criteria and considered probably related to E6201. During expansion (60-min infusion), ECG QT prolongation was reported as an AE (grade 3) by one (3.3%) patient, not meeting DLT criteria, but considered possibly related to E6201. No dose-related or mutation status-related trends in QT/QTc prolongation were observed.

Some ocular toxicity occurred at the E6201 160 and 320 mg/m^2^ once-weekly dose levels: increased lacrimation, blurred vision, visual impairment, and photophobia. All except visual impairment were considered possibly related to E6201, but were mild-to-moderate in severity. No new skin cancers were reported. In the expansion part, 1 patient (*BRAF*-WT 320 mg/m^2^ once-weekly) had grade 1 melanocytic nevus. Additionally, one patient (*BRAF*-mutated 320 mg/m^2^ once-weekly) had grade 5 metastatic malignant melanoma. Both events were considered unrelated to E6201. The incidence of cardiac disorders was low and there were no reports of cardiomyopathy. The safety profile for E6201 in this study contrasts with that of the oral MEK1/MEK2 inhibitor trametinib, which was associated with retinal pigment epithelial detachment, retinal vein occlusion, interstitial lung disease, skin toxicity, and cardiomyopathy in clinical trials (Mekinist (trametinib) tablets, for oral use [package insert]. (Novartis Pharmaceuticals Corporation, East Hanover, NJ, 2017)).

E6201 exposure appeared to be dose-related up to 320 mg/m^2^, with no incremental increase in exposure at higher doses. Achieved average plasma concentrations were well in excess of the half maximal inhibitory concentration (IC_50_) for inhibition of phosphor-ERK levels (IC_50_ = 2.08 nmol/L (1.09 ng/mL)), inhibition of cyclin D1 (IC_50_ = 4.8 nmol/L (1.87 ng/mL)) and inhibition of spontaneous secretion of IL-6 (IC_50_ = 10.5 nmol/L (4.09 ng/mL)) and IL-8 (IC_50_ = 21.8 nmol/L (8.19 mg/mL)).

During dose escalation, a PR was observed in a patient with *BRAF*-mutated papillary thyroid cancer (*KRAS*-WT) receiving E6201 480 mg/m^2^ once-weekly. As patients in part A of the study (dose-escalation phase) were not selected by *BRAF* mutational status (only three were confirmed with *BRAF*-mutated tumours), it was uncertain if the limited clinical response observed was related to *BRAF* status. During expansion, a PR occurred in 2 patients with *BRAF*-mutated melanoma (1 *KRAS*-WT and 1 of unknown *KRAS* status) and 1 with *BRAF*-WT, *KRAS*-WT melanoma; all three received E6201 320 mg/m^2^ once-weekly. Duration of response ranged from ≥2 cycles (*BRAF*-WT patient) to >40 cycles (both *BRAF*-mutated patients), suggesting that durable responses are possible with E6201. Of particular note, 1 patient with melanoma who received >40 cycles of therapy with E6201 also had a response in brain metastases during dose escalation, which suggests that E6201 may cross the blood–brain barrier. SD was observed in approximately one-third of patients during each part of the study. Most of these patients had SD for ≥4 treatment cycles, suggesting a durable response to E6201. The DCR for this part of the study was greater for patients with *BRAF*-mutated melanoma (43.5%) compared with *BRAF*-WT melanoma (28.6%), although any direct comparisons between the groups are unreliable due to the small numbers of *BRAF*-WT patients.

MEK inhibitors are a relatively recently developed therapeutic class. As two patients (both with *BRAF*-mutated melanoma and a best response of PR) have now received E6201 for ≥40 cycles, this study provides evidence of clinical benefit and some longer-term toxicity information. No safety concerns were reported for these patients. The favourable (if limited) response profile observed for E6201 administered once-weekly, despite its short half-life, provides justification for intermittent kinase inhibition as a valid alternative concept to continuous kinase inhibition. Another report of intermittent kinase inhibition was published in 2014.^19^

In conclusion, this study of E6201 in patients with advanced solid malignancies, including melanoma, suggests that an intermittent regimen of 320 mg/m^2^ by IV infusion once-weekly for the first 3 weeks of a 28-day cycle is a feasible and reasonably well-tolerated treatment approach. Additionally, we show preliminary evidence of clinical efficacy, confirming the potential of MEK inhibition and selective MEK1 inhibition, as a therapeutic strategy in cancer. E6201 is being further investigated in a phase 1/2 trial in patients with advanced haematologic malignancies with the *FLT3* mutation (www.clinicaltrials.gov, NCT02418000).

## Statement of translational relevance

The Raf/Ras/MEK/extracellular signal-related kinase signalling pathway plays a role in oncogenesis and tumour cell survival, and MEK inhibition is a potential therapeutic strategy in cancer. E6201 is a potent inhibitor of mitogen-activated protein kinase/extracellular signal-regulated kinase kinase-1 (MEK1) and mitogen-activated protein kinase/extracellular signal-regulated kinase kinase kinase-1 (MEKK1), as well as other kinases that have a role in cancer development. We report a phase 1 first-in-human study of E6201 in patients with advanced solid tumours, which was expanded to include patients with advanced melanoma. An intermittent regimen of E6201 320 mg/m^2^ once-weekly for the first 3 weeks of a 28-day cycle was feasible and reasonably well-tolerated in this patient population, with evidence of clinical efficacy including response in a melanoma patient with brain metastases. These findings confirm the potential of MEK inhibition, and selective MEK1 inhibition, as a therapeutic strategy in cancer.

## Electronic supplementary material

Supplementary Material

Figure S1

## Availability of data and material

The datasets generated during and/or analysed during the current study are on file with Eisai and not publicly available.
