# Supplementary material for: Safety, pharmacokinetics, and preliminary efficacy of E6201 in patients with advanced solid tumours, including melanoma: results of a phase 1 study
Source: Br J Cancer. 2018 Jun 5;118(12):1580–5. doi: 10.1038/s41416-018-0099-5 (PMC6008465; doi:10.1038/s41416-018-0099-5)
Supplement: Supplementary file 1 — Supplementary Material [file 41416_2018_99_MOESM1_ESM.docx]

**Supplementary Material**

**Key exclusion criteria**

Patients excluded from Part A included those with primary or metastatic central nervous system (CNS) tumors (unless treated and stable), those with a history of malignancy other than the present diagnosis (except treated non-melanoma skin cancer or carcinoma in situ of the cervix). Patients with prior primary CNS tumors had to be in complete remission and off therapy for that disease for a minimum of 5 years. For Part B, patients with active malignancy other than the present diagnosis within the past 24 months (except treated non-melanoma skin cancer or carcinoma in situ of the cervix) were excluded, although treated or untreated CNS metastases from a primary melanoma were allowed. For both Parts A and B, patients could not have undergone prior surgery (specified as prior major surgery in Part B), or received radiotherapy, chemotherapy, biologic therapy, or investigational agents within 4 weeks prior to the first E6201 infusion or prior immunotherapy, or hormonal (except, for Part B, hormonal treatment for prostate cancer and adjuvant therapy in breast cancer) or molecular-targeted therapy within 2 weeks prior. Those with a QT interval corrected for rate (QTc) >450 msec (on the electrocardiogram obtained at screening using the Fridericia method for QTc analysis), or a recent history of cardiac events or dysfunction were also excluded.

| **Supplementary Table S1.** Incidence of the most common treatment-emergent adverse events (occurring in ≥15% of patients overall in Parts A or B; safety population) | | | | | | | | | | | | | | | | |
| --- | --- | --- | --- | --- | --- | --- | --- | --- | --- | --- | --- | --- | --- | --- | --- | --- |
| **Part A** | | | | | | | | | | | | | | | | |
| **Category, n (%)** | **Initial E6201 dose group (mg/m^2^)** | | | | | | | | | | | | | | **Total (N=25)** | |
|  | **20 (n=4)** | | **40 (n=3)** | | **80 (n=3)** | | **160 (n=3)** | | **320 (n=7)** | | **400 (n=1)** | | **480 (n=4)** | |  |  |
|  | **All** | **G≥3** | **All** | **G≥3** | **All** | **G≥3** | **All** | **G≥3** | **All** | **G≥3** | **All** | **G≥3** | **All** | **G≥3** | **All** | **G≥3** |
| Nausea | 1 (25.0) | 0 | 1 (33.3) | 0 | 0 | 0 | 1 (33.3) | 0 | 5 (71.4) | 1 (14.3) | 0 | 0 | 1 (25.0) | 0 | 9 (36.0) | 1 (4.0) |
| Constipation | 1 (25.0) | 0 | 0 | 0 | 1 (33.3) | 0 | 1 (33.3) | 0 | 2 (28.6) | 0 | 0 | 0 | 2 (50.0) | 0 | 7 (28.0) | 0 |
| Fatigue | 1 (25.0) | 0 | 0 | 0 | 0 | 0 | 1 (33.3) | 0 | 3 (42.9) | 1 (14.3) | 0 | 0 | 2 (50.0) | 0 | 7 (28.0) | 1 (4.0) |
| Hypokalemia | 1 (25.0) | 0 | 1 (33.3) | 0 | 0 | 0 | 0 | 0 | 2 (28.6) | 0 | 1 (100) | 1 (100) | 2 (50.0) | 0 | 7 (28.0) | 1 (4.0) |
| Vomiting | 0 | 0 | 1 (33.3) | 0 | 0 | 0 | 1 (33.3) | 0 | 4 (57.1) | 0 | 0 | 0 | 1 (25.0) | 0 | 7 (28.0) | 0 |
| Abdominal pain | 1 (25.0) | 0 | 1 (33.3) | 0 | 0 | 0 | 1 (33.3) | 1 (33.3) | 2 (28.6) | 1 (14.3) | 0 | 0 | 1 (25.0) | 0 | 6 (24.0) | 2 (8.0) |
| Decreased appetite | 1 (25.0) | 0 | 0 | 0 | 0 | 0 | 1 (33.3) | 0 | 2 (28.6) | 1 (14.3) | 0 | 0 | 1 (25.0) | 0 | 5 (20.0) | 1 (4.0) |
| Peripheral edema | 1 (25.0) | 0 | 0 | 0 | 1 (33.3) | 0 | 1 (33.3) | 0 | 1 (14.3) | 1 (14.3) | 0 | 0 | 0 | 0 | 4 (16.0) | 1 (4.0) |

| **Part B** | | | | | | | | |
| --- | --- | --- | --- | --- | --- | --- | --- | --- |
| **Category, n (%)** | **Initial E6201 dose group** | | | | | | **Total (N=30)** | |
|  | ***BRAF*-mutated 320 mg/m^2^**  **once weekly (n=20)^a^** | | ***BRAF*-mutated 160 mg/m^2^ twice weekly (n=3)** | | ***BRAF*-WT 320 mg/m^2^ once weekly (n=7)^b^** | |  |  |
|  | **All** | **G≥3** | **All** | **G≥3** | **All** | **G≥3** | All | **G≥3** |
| Fatigue | 7 (35.0) | 0 | 0 | 0 | 4 (57.1) | 0 | 11 (36.7) | 0 |
| Nausea | 7 (35.0) | 0 | 1 (33.3) | 1 (33.3) | 2 (28.6) | 0 | 10 (33.3) | 1 (3.3) |
| Vomiting | 5 (25.0) | 0 | 1 (33.3) | 1 (33.3) | 1 (14.3) | 0 | 7 (23.3) | 1 (3.3) |
| Anemia | 1 (5.0) | 0 | 1 (33.3) | 0 | 3 (42.9) | 1 (14.3) | 5 (16.7) | 1 (3.3) |
| Constipation | 3 (15.0) | 0 | 0 | 0 | 2 (28.6) | 0 | 5 (16.7) | 0 |
| Cough | 3 (15.0) | 0 | 1 (33.3) | 0 | 1 (14.3) | 0 | 5 (16.7) | 0 |
| Decreased appetite | 3 (15.0) | 0 | 0 | 0 | 2 (28.6) | 0 | 5 (16.7) | 0 |
| Muscular weakness | 2 (10.0) | 0 | 1 (33.3) | 0 | 2 (28.6) | 0 | 5 (16.7) | 0 |
| Peripheral edema | 2 (10.0) | 0 | 1 (33.3) | 0 | 2 (28.6) | 1 (14.3) | 5 (16.7) | 1 (3.3) |
| Pain in extremity | 2 (10.0) | 0 | 1 (33.3) | 0 | 2 (28.6) | 1 (14.3) | 5 (16.7) | 1 (3.3) |
| Pyrexia | 4 (20.0) | 0 | 0 | 0 | 1 (14.3) | 0 | 5 (16.7) | 0 |

^a^ 1 Patient died from previously diagnosed metastatic malignant melanoma that was unrelated to E6201. No new skin cancers were reported in any patients in the study.

^b^ 1 Patient had a melanocytic nevus (unrelated to E6201).

G: Grade.

| **Supplementary Table S2.** Mean (%CV) pharmacokinetic parameters following weekly E6201 administration. | | | | | | | |
| --- | --- | --- | --- | --- | --- | --- | --- |
| **PK Parameter** | **Cycle 1 Day 1** | | | | | | |
|  | **20 mg/m^2^ (n=4)** | **40 mg/m^2^ (n=3)** | **80 mg/m^2^ (n=3)** | **160 mg/m^2^ (n=3)** | **320 mg/m^2^ (n=7)** | **400 mg/m^2^ (n=1)** | **480 mg/m^2^ (n=4)** |
| C_max_ (ng/mL) | 552 (50) | 870 (70) | 3350 (149) | 2740 (51) | 14800 (73) | 19500 (--) | 8070 (55) |
| AUC_(0-t)_ (ng·h/mL) | 1650 (218) | 2410 (201) | 2520 (63) | 2770 (36) | 10400 (26) | 13500 (--) | 10200 (56) |
| T_max_^a^ (h) | 0.5 (0.25–0.5) | 0.5 (0.5–0.5) | 0.5 (0.25–0.5) | 0.5 (0.25–0.5) | 0.25 (0.25–0.5) | 0.25 (0.25–0.25) | 0.375 (0.25–0.5) |
| t_½_ (h) | 1.96^b^ (--) | 2.70^b^ (--) | 2.79 (--) | 3.98 (--) | 4.00 (--) | 6.50 (--) | 5.97 (--) |
| CL (L/h) | 110^b^ (2) | 103^b^ (25) | 68.7 (60) | 119 (50) | 57.2 (22) | 52.8 (--) | 101 (44) |
| V_ss_ (L) | 135^b^ (28) | 164^b^ (21) | 135 (100) | 216 (55) | 89.1 (36) | 80.1 (---) | 275 (42) |
| CL_R_ (mL/h) | 10.0 (188) | 1.65 (--) | 28.9 (54) | 89.7 (299) | 60.0 (659) | 111 (--) | 137 (128) |
| f_e_ (%) | 0.0125 (42) | 0 (--) | 0.04 (26) | 0.0567 (186) | 0.109 (56) | 0.21 (--) | 0.143 (96) |
| **Cycle 1 Day 15** | | | | | | | |
| **PK Parameter** | **20 mg/m^2^ (n=3)** | **40 mg/m^2^ (n=3)** | **80 mg/m^2^ (n=3)** | **160 mg/m^2^ (n=3)** | **320 mg/m^2^ (n=6)** | **400 mg/m^2^ (n=1)** | **480 mg/m^2^ (n=4)** |
| C_max_ (ng/mL) | 895 (228) | 767 (78) | 2590 (154) | 5430 (120) | 18000 (64) | 21600 (--) | 12200 (88) |
| AUC_(0-t)_ (ng·h/mL) | 1420 (250) | 2090 (169) | 2540 (66) | 4300 (32) | 11100 (13) | 10500 (--) | 11300 (73) |
| T_max_^a^ (h) | 0.5 (0.25–0.5) | 0.5 (0.25–0.5) | 0.25 (0.25–0.5) | 0.25 (0.25–0.5) | 0.25 (0.25–0.5) | 0.25 (0.25–0.25) | 0.375 (0.25–0.5) |
| t_½_ (h) | 1.58^c^ (--) | 1.88 ^b^ (--) | 5.55 (--) | 4.20 (--) | 6.83 (--) | -- | 4.78 (--) |
| CL (L/h) | 184^c^ (--) | 91^b^ (47) | 76.2 ^b^ (67) | 71.2 (18) | 49.8 (18) | -- | 82 (38) |
| V_ss_ (L) | 324 ^c^ (--) | 115^b^ (27) | 148 ^b^ (146) | 110 (74) | 111 (69) | -- | 217 (84) |

^a^ Median (max–min).

^b^ n=2.

^c^ n=1.

f_e_: fraction of drug excreted in urine; CL_R_: renal clearance; C_max_: maximum observed plasma concentration; AUC_(0-t)_: area under the plasma concentration time curve from time zero to the last quantifiable sample; t_1/2_: terminal elimination half-life; T_max_: time of maximum observed plasma concentration; CL: total clearance; V_ss_: steady-state volume of distribution.

| **Supplementary Table S3.** Best overall response (safety population) | | | | | | | | |
| --- | --- | --- | --- | --- | --- | --- | --- | --- |
| **Part A** | | | | | | | | |
| **Category (%)** | **Initial E6201 dose group (mg/m^2^)** | | | | | | | **Total (N=25)** |
|  | **20  (n=4)** | **40  (n=3)** | **80**  **(n=3)** | **160**  **(n=3)** | **320**  **(n=7)** | **400**  **(n=1)** | **480**  **(n=4)** |  |
| Complete response (CR) | 0 | 0 | 0 | 0 | 0 | 0 | 0 | 0 |
| Partial response (PR) | 0 | 0 | 0 | 0 | 0 | 0 | 1 (25.0)^a^ | 1 (4.0) |
| Stable disease (SD) | 1 (25.0) | 2 (66.7)^b^ | 1 (33.3)^c^ | 0 | 1 (14.3) | 1 (100)^d^ | 2 (50.0)^e^ | 8 (32.0) |
| Progressive disease (PD) | 3 (75.0) | 1 (33.3) | 2 (66.7) | 2 (66.7) | 4 (57.1) | 0 | 0 | 12 (48.0) |
| Not evaluable (NE) | 0 | 0 | 0 | 1 (33.3) | 1 (14.3) | 0 | 0 | 2 (8.0) |
| Overall response rate (CR+PR) | 0 | 0 | 0 | 0 | 0 | 0 | 1 (25.0) | 1 (4.0) |
| Disease control rate (CR+PR+SD) | 1 (25.0) | 2 (66.7) | 1 (33.3) | 0 | 1 (14.3) | 1 (100) | 3 (75.0) | 9 (36.0) |

^a^ Patient with papillary thyroid cancer (*BRAF*-mutated), PR x 4 cycles.

^b^ 1Patient with colon cancer (*BRAF*-WT), SD x 6 cycles; 1 patient with biliary duct cancer (*BRAF*-WT), SD x 4 cycles.

^c^ Patient with uveal melanoma (*BRAF*-WT) x 15 cycles.

^d^ Patient with gastric carcinoid (*BRAF* status unknown), SD x 4 cycles.

^e^ Includes 1 patient with papillary thyroid cancer (*BRAF*-WT), SD x 4 cycles.

| **Part B** | | | | |
| --- | --- | --- | --- | --- |
|  | **Initial E6201 dose group** | | | **Total**  **(N=30)** |
|  | ***BRAF*-mutated**  **320 mg/m^2^ once weekly (n=20)** | ***BRAF*-mutated**  **160 mg/m^2^ twice weekly (n=3)** | ***BRAF*-WT**  **320 mg/m^2^ once weekly**  **(n=7)** |  |
| Complete response (CR), n (%) | 0 | 0 | 0 | 0 |
| Partial response (PR), n (%) | 2 (10.0)^a^ | 0 | 1 (14.3)^b^ | 3 (10.0) |
| Stable disease (SD), n (%) | 7 (35.0)^c^ | 1 (33.3)^d^ | 1 (14.3)^e^ | 9 (30.0) |
| Progressive disease (PD), n (%) | 6 (30.0) | 1 (33.3) | 2 (28.6) | 9 (30.0) |
| Not evaluable (NE), n (%) | 1 (5.0) | 0 | 1 (14.3) | 2 (6.7) |
| Overall response rate (CR+PR), n (%) | 2 (10.0) | 0 | 1 (14.3) | 3 (10.0) |
| Disease control rate (CR+PR+SD), n (%) | 9 (45.0) | 1 (33.3) | 2 (28.6) | 12 (40.0) |
| Median (95% CI) duration of SD, days^f^ | 73.0 (29.0–166.0) | 138.0 (29.0–138.0) | NE | - |
| Median (95% CI) duration of PFS, days^f^ | 73.0 (26.0–166.0) | 83.5 (29.0–138.0) | NE | - |

^a^ >40 cycles.

^b^ >2 cycles.

^c^ Includes 5 patients x ≥4 cycles.

^d^ >4 cycles.

^e^ x 4 cycles.

^f^ Kaplan-Meier estimate.

NE: not evaluable.

**Supplementary Figure S1.** Changes in tumor size during the study.
